# The sympathy of two pendulum clocks: beyond Huygens’ observations

**DOI:** 10.1038/srep23580

**Published:** 2016-03-29

**Authors:** Jonatan Peña Ramirez, Luis Alberto Olvera, Henk Nijmeijer, Joaquin Alvarez

**Affiliations:** 1 Center for Scientific Research and Higher Education at Ensenada (CICESE), C.P. 22860, Ensenada, B.C., México; 2 Relojes Centenario, Department of Production Engineering, C.P. 73310, Zacatlan, Puebla, México; 3 Eindhoven University of Technology, Department of Mechanical Engineering, P.O. Box 513, Eindhoven, The Netherlands

## Abstract

This paper introduces a modern version of the classical Huygens’ experiment on synchronization of pendulum clocks. The version presented here consists of two monumental pendulum clocks—ad hoc designed and fabricated—which are coupled through a wooden structure. It is demonstrated that the coupled clocks exhibit ‘sympathetic’ motion, i.e. the pendula of the clocks oscillate in consonance and in the same direction. Interestingly, when the clocks are synchronized, the common oscillation frequency decreases, i.e. the clocks become slow and inaccurate. In order to rigorously explain these findings, a mathematical model for the coupled clocks is obtained by using well-established physical and mechanical laws and likewise, a theoretical analysis is conducted. Ultimately, the sympathy of two monumental pendulum clocks, interacting via a flexible coupling structure, is experimentally, numerically, and analytically demonstrated.

In an era when science relied heavily on observation, experimentation, and reflection, the Dutch scientist Christiaan Huygens made a serendipitous discovery: two of his recently invented pendulum clocks—which were hanging from a common wooden beam placed at the top of two chairs—were showing an ‘odd sympathy’. Namely, the pendula of the clocks were oscillating in perfect consonance but in opposite directions, i.e. the clocks were *synchronized* in anti-phase. He reported this odd phenomenon first to R. F. de Sluse, on February 22, 1665 and two days later to his father and to a member of the Royal Society of London^1^,^2^.

Although at that time Huygens did not have the proper mathematical tools for explaining his observations—differential calculus had not been invented yet—he managed to find the mechanism responsible for the sympathy in his clocks: (the small vibrations of) the wooden bar on which the clocks were hanging.

For some reason, the sympathetic motion of pendulum clocks discovered by Huygens, hereinafter called *Huygens’ synchronization*, did not attract the attention of the scientific community at that time. In fact, a ‘hot topic’ in those days was the problem of finding the longitude coordinate at sea. However, in 1739, the English clockmaker John Ellicot reported an odd phenomenon: two pendulum clocks placed sideways were interacting in such a way that the oscillations of one pendulum clock were quenched^3^,^4^. Latter, in 1873, the English astronomer William Ellis, noticed a sympathetic behaviour on two clocks that were placed on a common wooden stand: during several consecutive days, the pendula of the clocks were oscillating in harmony such that one pendulum was swinging to the left while the other pendulum was swinging to the right. Interestingly, Ellis attempted a ‘network’ experiment using 9 pendulum clocks. In this case, however, the previously observed harmony disappeared^5^. Unfortunately, neither Ellicot nor Ellis made a reference to the work of Huygens.

At the beginning of the 20^th^ century, D.J. Korteweg made the first theoretical attempt to explain Huygens’ observations. Specifically, Korteweg derived a linear model, neglecting damping and driving forces in the pendula. With his model, Korteweg envisioned that ‘other kinds of sympathy’ may be possible^6^. Besides this, the sympathy of coupled clocks was still considered as a fairly difficult problem among scientists and clockmakers at that time^7^. In fact, a Nature paper of 1911, reported: “it is apparently beyond human ingenuity to produce two clocks which will go together for one week”^8^. In fact, the same paper refers to an experiment, due to Mr. R. L. Jones of Chester, in which the pendula of a group of clocks were forced to beat ‘in sympathy’ by means of a regulator.

In the last years, Huygens’ synchronization has become a relevant topic among scientists and researchers. By designing novel experimental platforms^9^,^10^,^11^,^12^,^13^ and/or by conducting theoretical analyzes^14^,^15^,^16^,^17^,^18^,^19^,^20^,^21^,^22^,^23^,^24^, further understanding about the exciting phenomenon described by Huygens has been obtained. In particular, the aforementioned studies somehow convey the same message: the key element in Huygens’ setup of pendulum clocks is the coupling structure and its mechanical properties.

Nevertheless, Huygens’ synchronization is still an open problem. This claim may be surprising, specially if one considers the fact that the behavior associated to pairs of coupled oscillators has been extensively and exhaustively studied and nowadays, the focus is not on pairs of oscillators but rather in networks of oscillators. Hence, it seems necessary to justify the need of further studies regarding Huygens’ synchronization and to clearly establish the current challenges. In consonance with this, consider Fig. 1, which presents a schematic representation of Huygens’ experiment. The left side of the yellow rectangle shows the two main components namely, the coupling structure (orange circle), and the pendulum clocks (blue circle). By adding these two components, one obtains the experimental platform used by Huygens^25^. On the right side of the figure, the ‘key ingredients’ necessary for a thorough understanding of Huygens’ synchronization are presented: a proper modelling of the coupling structure and a proper model for the pendulum clocks.

Although the design and construction of a mechanical clock is a highly non trivial task, the mathematical modelling of the clock can be achieved relatively ‘easy’. In fact, many authors agree that a second order nonlinear differential equation suffices for describing the dynamic behaviour of a pendulum clock. Perhaps, some difficulties may arise when modelling the escapement mechanism of the clocks. However, this may be circumvented by introducing nonlinear terms describing or rather mimicking the operation of the escapement.

The real challenge about Huygens’ synchronization is ‘enclosed’ in the orange circles in Fig. 1. It should be noted that a proper mathematical model has not been derived yet. Current models are an oversimplification of the real Huygens’ system, in part because the coupling structure has been assumed to be rigid, see e.g.^23^,^26^. However, the coupling structure used by Huygens, a wooden beam on the top of two chairs, is in fact a flexible body, which necessarily needs to be modeled by using a partial differential equation with suitable boundary conditions. As far as is known, such model does not exist in the literature, although there exist preliminary works on this direction, see e.g.^27^,^28^. Likewise, it is still necessary to determine ‘all’ possible limit solutions, besides synchronous solutions, in Huygens’ system of coupled pendulum clocks cf.^24^.

In this regard, the present contribution aims to add to the current knowledge about Huygens’ synchronization phenomenon. In particular, a novel experimental platform, which is reminiscent of Huygens’ synchronization experiment, is presented. The platform consists of two monumental clocks, which are mounted on a common wooden table. Although preliminary attempts have been done by using either metronomes^9^,^10^,^11^,^29^ or small commercial clocks^12^,^30^, as far as is known, this is the first time that Huygens’ experiment is reproduced by using monumental clocks. Additionally, we have discovered that the oscillation frequency of the synchronized clocks is indeed affected. Furthermore, an improved model, or rather a ‘more natural’ model regarding current models, is introduced and finally, by using the theory of piece-wise linear systems, the occurrence of synchronized motion in the clocks is analytically studied.

In summary, this work presents insightful results—i.e. novel experiments, an improved mathematical model, and a rigorous theoretical analysis—related to Huygens’ synchronization phenomenon.

## Results

As a first step, an experimental study is presented. The outcome of the experiments has revealed that the monumental pendulum clocks introduced here exhibit in-phase synchronized motion. This result seems to be contrary to Huygens observations who, to the best of our knowledge, only observed anti-phase synchronization in his setup of pendulum clocks, although very likely he was aware of the possibility of observing in-phase synchronized motion in his clocks. Furthermore, the experiments also show that when synchronized, the pendulum clocks become ‘slow’, i.e. their oscillation frequency decreases. Next, in order to obtain rigorous explanations for these results, a mathematical model, which takes into account the flexibility of the coupling structure, i.e. the flexibility of the wooden table on which the clocks are mounted, is derived by using the Finite Element method. Likewise, it is shown that under some mild assumptions, Huygens’ system of coupled clocks can be considered as a piece-wise linear system. Finally, analytic conditions for the existence of stable synchronized motion in the coupled clocks are provided.

### Experimental results

**Figure 1:**
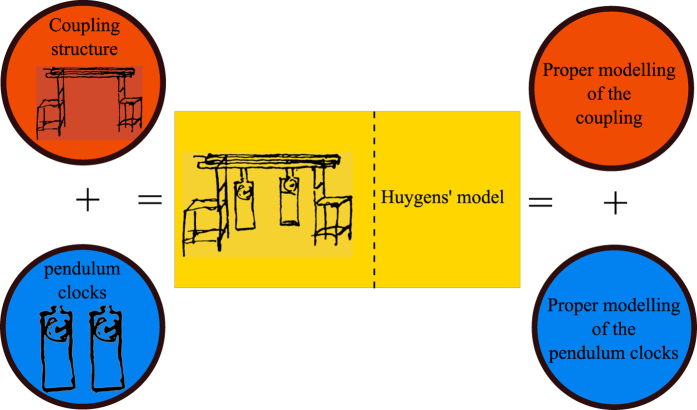
Huygens’ setup (see^1^,^25^).

**Figure 2:**
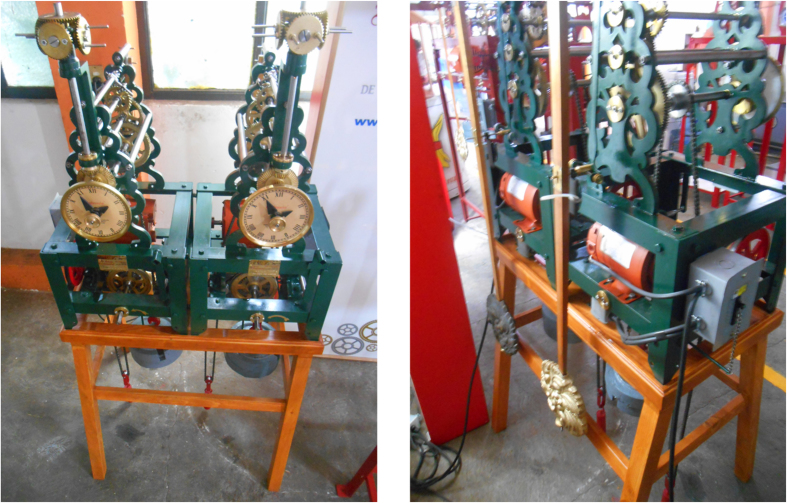
Experimental setup at Relojes Centenario.

The experimental platform used in this study is depicted in Fig. 2. It consists of two monumental clocks placed on the top of a wooden structure. The clocks are ad hoc designed and constructed, *as identical as possible*, by the clocks factory *Relojes Centenario*, located at Zacatlán, Puebla, México, who is the industrial partner in the research reported here. Each clock has a pendulum, which consists of a metallic mass attached to the lower end of a wooden rod. The weight of the pendulum mass is 5 [kg] and the length of the rod is 0.99 [m]. At the ‘heart’ of each clock there is an anchor escapement mechanism, which is driven by suspended weights. On the other hand, the structure on which the clocks are placed is made of pine wood. The mechanical and geometrical properties for the structure and for the clocks, are provided in Tables 1 and 2, respectively. At this point, it is worth mentioning that the design of the coupling structure is inspired by our previous theoretical work^24^. Additionally, it should be noted that the experimental setup depicted in Fig. 2 is slightly different than the one used by Huygens. In our case, the clocks are supported on the structure, whereas in Huygens’ experiment the clocks were hanging from the structure, see Fig. 1, but in both structures flexibility, i.e. elastic deformation of the material (wood), is present.

The experiments are described as follows. The pendula of the clocks are initialized from opposite positions. However, after a long transient behaviour of approximately 30 minutes, the pendula reached consonance such that the pendula oscillate in the same direction and at the same frequency and amplitude, i.e. the pendula of the clocks are synchronized in-phase. Once the clocks are synchronized, they remain in this state as long as there is potential energy stored in the weights driving the escapement mechanism.

**Figure 3:**
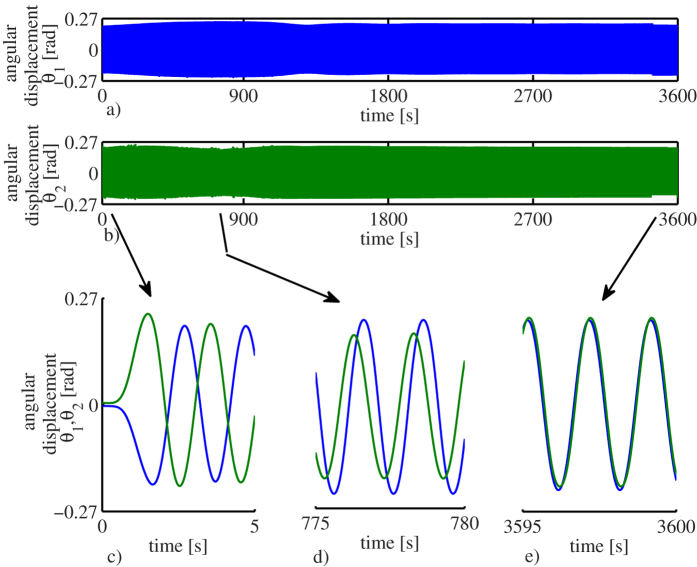
Experimental results for an experiment lasting one hour. (**a**) Angular displacement of pendulum one. (**b**) Angular displacement of pendulum two. (**c**) The clocks are initialized close to anti-phase motion. (**d**) Pendulum one gains amplitude, whereas pendulum two loses amplitude and the phase difference decreases. (**e**) After a long transient behaviour, the pendula synchronize in-phase.

The obtained experimental results, for an experiment lasting 1 hour, are presented in Fig. 3. The complete time series are presented in Fig. 3(a,b), where the blue time series corresponds to the angular displacement of pendulum one, whereas the green time series denotes the angular displacement corresponding to pendulum two. Although from these figures the onset of synchronization is not clear, it becomes evident that ‘something’ happens in the interval *t* ∈ [300, 1100] [s]: the first pendulum clock (blue line) gains amplitude, whereas the second pendulum clock (green line) loses amplitude.

In order to have a a better insight into the experimental results, Fig. 3(c–e), which are snapshots corresponding to Fig. 3(a,b), are presented. The first 5 seconds of the experiment are depicted in Fig. 3(c), from which the initial anti-phase motion of the pendulum clocks becomes evident. Then, Fig. 3(d) shows the time evolution of the angular displacements of the clocks in the interval *t* ∈ [775, 780] [s]. From this figure it is clear that the amplitude corresponding to the angular displacement of pendulum one (blue line) is larger than the amplitude of pendulum two (green line) around that interval of time. Finally, a snapshot to the last 5 seconds of the experiment, see Fig. 3(e), shows that the clocks are indeed synchronized in-phase.

**Figure 4:**
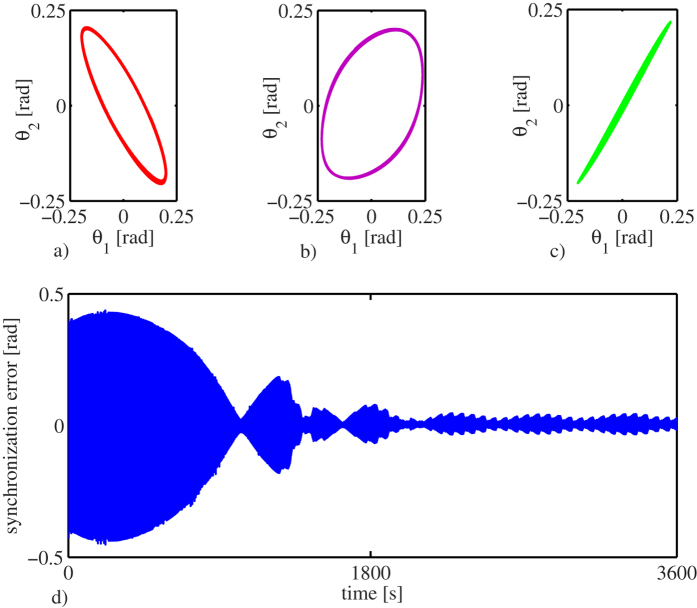
Experimental results. (**a**–**c**) Projections of the dynamic behaviour of the clocks onto the (*θ*_1_, *θ*_2_)-plane corresponding to the time series depicted in Fig. 3(c–e), respectively. (**d**) Synchronization error 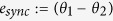.

In order to further illustrate the onset of synchronization, the projections of the dynamic behaviour of the clocks onto the (*θ*_1_, *θ*_2_)-plane, are presented in Fig. 4(a–c), where 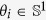, *i* = 1, 2, denotes the angular displacement of pendulum *i*. Note that Fig. 4(a–c) correspond to the time series presented in Fig. 3(c–e), respectively. From these projection plots the (close to) anti-phase startup, the transient behaviour, and the ‘steady’ in-phase synchronized motion, are clearly seen.

Finally, the synchronization error, which is defined as the difference between the angular displacement 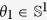 of pendulum one and the angular displacement 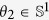 of pendulum two, i.e. 
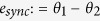
, is presented in Fig. 4(d). Note that at the beginning the error is large, due to the fact that the pendula are initialized from opposite directions. However, after a long transient behaviour, the error ‘almost’ vanishes. At this point is worth to note that in the error time series depicted in Fig. 4(d), there are instants at which the error seems to decrease to zero but ‘suddenly’ grows and decreases again.

This is explained as follows. At the instants where the error seems to grow, an electrical motor, rewinding the suspended weights driving the escapement, is activated for a few seconds. Hence, the vibration of the motor induces a disturbance to the pendula and as a consequence, the synchronized motion is momentarily disturbed. However, once the ‘steady’ motion of the clocks is achieved (after half an hour), the influence of the vibrations of the motor on the clocks is negligible, and consequently the clocks are synchronized in-phase for the rest of the experiment. This is clearly seen in Fig. 4(d). One cannot expect the synchronization error to be zero, since there are unavoidable mismatches in the clocks. What we are observing in the experiment, and in general in any synchronization experiment, is a so-called practical synchronization^31^,^32^.

**Figure 5:**
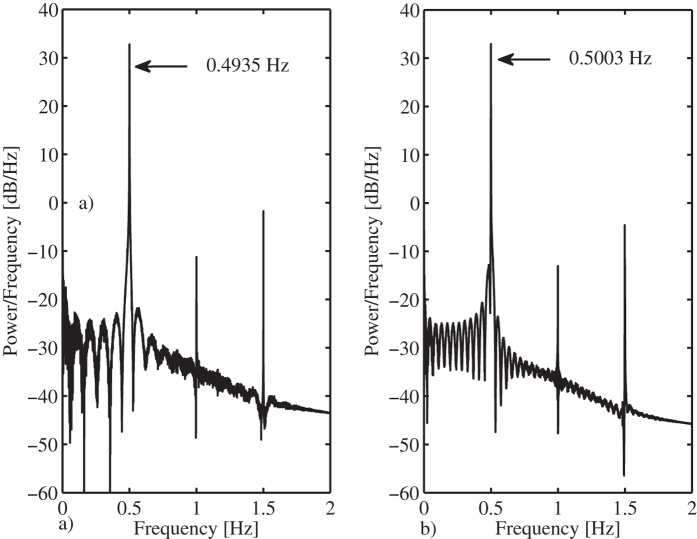
Oscillation frequency. (**a**) Coupled and synchronized clocks. (**b**) Uncoupled clock.

On the other hand, it has been found that, when synchronized, the clocks become slow, i.e. the oscillation frequency of the synchronized clocks is lower than the oscillation frequency of an uncoupled clock. This is depicted in Fig. 5. The right panel depicts the oscillation frequency for an uncoupled clock, which is 0.5003 [Hz], whereas the left panel shows the oscillation frequency for the coupled clocks, which is 0.4935 [Hz]. In other words, the coupled clocks will lose 47.34 [s] per hour or, equivalently, 1136.16 [s] per day!

This result coincides with our previous theoretical work^22^, where it has been shown that the stiffness of the coupling structure plays a key role in determining the oscillation frequency of the synchronized clocks, namely, for a coupling structure with ‘low’ stiffness, the clocks will oscillate at a frequency higher than the oscillation frequency corresponding to an uncoupled clock and for a coupling structure with relatively large stiffness, the oscillation frequency will be lower than the oscillation frequency of an uncoupled clock, just like in the experiment with monumental clocks presented here. Moreover, the change in the oscillation frequency may also be associated to the loss of energy in the clocks, via the damped coupling structure, see e.g.^9^,^29^.

As a final remark, it should be noted that for the sake of easy explanation, only one experiment has been reported here. However, we want to stress the fact that the experiment has been repeated several times (more than 100) and in all the trials the results coincide with the results presented in Figs 3, 4, 5, i.e. the experiment is reproducible. In particular, in such exhaustive experimental analysis (not reported here), different initial conditions have been used—including the case of starting the pendula of the clocks in anti-phase motion. It has been found that for all the considered initial conditions, the pendula always converge to the in-phase synchronized motion. Likewise, for the case where the clocks are running synchronized in-phase, if a small perturbation is applied to one pendulum (for example giving a push to the pendulum in the opposite direction of motion), the in-phase synchronized motion is restored after the effect of the perturbation vanishes.

#### Intermezzo: The Dutch National Science Quiz

**Figure 6:**
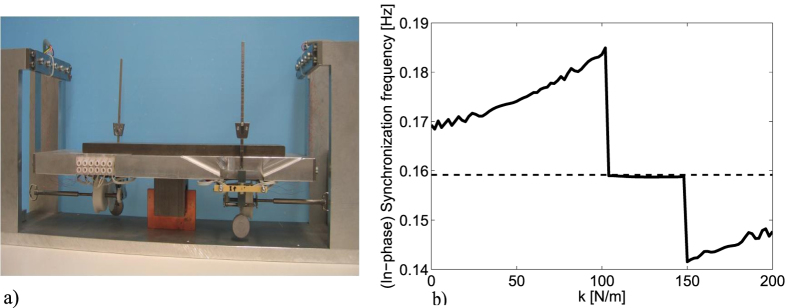
(**a**) Nijmeijer’s setup, see e.g.^11^. (**b**) Oscillation frequency of two coupled metronomes as a function of the stiffness.

In December 2012, during the Dutch National Science Quiz, organized by the Netherlands Organisation for Scientific Research (NWO) and the Dutch public broadcaster VPRO, 15 science-related questions were posed to to the public, one of them read: Consider a pair of metronomes, with slightly different frequencies, mounted on a platform elastically attached to a fixed support. The platform can move only on the horizontal axis. Suppose that after some time the metronomes synchronize, What is the synchronization frequency? There were 3 possible answers: a) The oscillation frequency of the synchronized metronomes is lower than the average of frequencies corresponding to each uncoupled metronome. b) The oscillation frequency is the average of the oscillation frequencies of each uncoupled metronome. c) The oscillation frequency of the synchronized metronomes is higher than the average of frequencies corresponding to each uncoupled metronome. For the answer, we prepared an experiment using the Nijmeijer’s setup, see Fig. 6(a). The experiment, which was broadcasted to the Dutch audience, revealed that the answer is *A*). The answer is rather tricky as the correct answer depends on the stiffness coefficient of the spring of the platform (as well as various other mechanical characteristics of the platform and metronomes). This fact has been rigorously proved in ref. 22, where it has been shown that the oscillation frequency, existence, and stability of the in-phase solution—the solution where the metronomes oscillate in consonance at the same frequency, and amplitude and zero phase difference—are strongly determined by the spring stiffness of the platform, as depicted in Fig. 6(b), from which it is clear that depending on the value of the stiffness coefficient *k*, the oscillation frequency of the synchronous solution is above or below the oscillation frequency of the uncoupled metronomes (horizontal dotted line). In the interval where the oscillation frequency remains constant, independently of the value of *k*, the in-phase solution becomes unstable and instead, the metronomes synchronize in anti-phase. Clearly, it is almost impossible to find the correct answer to the aforementioned quiz at short notice and without a careful reasoning. Note, however, that the results obtained with the Nijmeijer’s setup (metronomes) coincide with the results presented in this manuscript (monumental clocks), i.e. in both cases the oscillators, either metronomes or pendulum clocks, show in-phase synchronized motion and become slow.

### Mathematical model and simulation results

In order to explain the experimental results presented in the previous section, a mathematical model describing the dynamic behaviour of the experimental setup of Fig. 2 is obtained.

**Figure 7:**
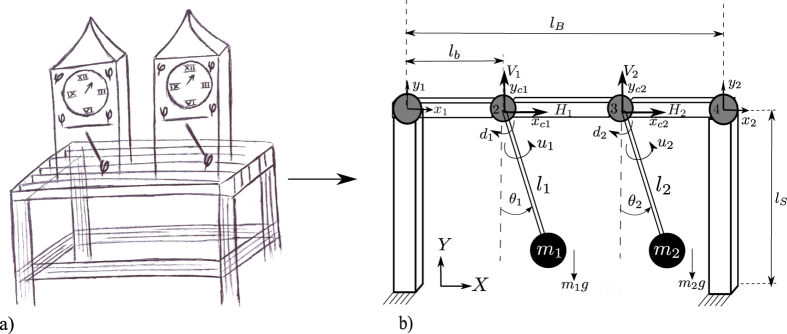
(**a**) Schematic drawing of the experimental setup depicted on Fig. 2. (**b**) Idealized schematic model. The structure has been divided into 5 pieces (white bars, 2 vertical and 3 horizontal) interconnected via 4 nodes (gray circles). Each clock has been modelled as a driven and damped pendulum.

First, the wooden coupling structure is modelled by using the Finite Element (FE) method^33^. In this way, the flexibility of the structure (bending and elongation) is taken into account^24^. The FE formalism requires to divide the structure into finite pieces, i.e. to discretize the spatial variable, with the final aim of getting a finite set of ordinary differential equations (ODEs). Figure 7 shows the resulting discretized structure, which consists of 5 beam elements (white bars, 2 vertical and 3 horizontal) interconnected through 4 nodes (gray circles). Each element is modelled using Euler beams. The vertical elements have length *l*_*S*_, whereas the horizontal elements have length *l*_*b*_. At each node, there are two degrees of freedom namely, translational and vertical motions. Consequently, the model describing the dynamical behaviour of the structure will consist of 8 ODEs.

As a second step, each clock is modelled as a driven and damped pendulum. In particular, it is assumed that each pendulum consists of a point mass of mass *m*_*i*_ attached to the lower end of a massless rod of length *l*_*i*_, for *i* = 1, 2. A further assumption is related to the attachment of the pendula to the structure. As is evident from Fig. 7, it is assumed, without loss of generality, that the pendula are directly attached to the structure by means of a revolute joint with viscous and linear damping. The damping coefficient is denoted by *d*_*i*_ [Nms/rad]. Likewise, the metallic case of each clock has been modelled as a point mass added to the node at which the clock is connected. Finally, the escapement mechanism in each clock is replaced by a suitable ‘escapement’ input *u*_*i*_, *i* = 1, 2, to be designed latter.

The aforementioned modelling process yields to the following (idealized) equations of motion:


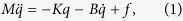



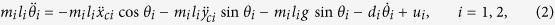


where *g* [m/s^2^] is the gravitational acceleration and *q* = [*x*_1_ *y*_1_ *x*_*c*1_ *y*_*c*1_ *x*_*c*2_ *y*_*c*2_ *x*_2_ *y*_2_]^*T*^ is the state vector. The state variables *x*_1_, *x*_2_ and *y*_1_, *y*_2_ denote the translational and vertical displacements, at node 1 and 4, respectively. Likewise, the state variables *x*_*c*1_, *x*_*c*2_ and *y*_*c*1_, *y*_*c*2_ describe the translational and vertical displacements of the nodes 2 and 3, respectively, at which the pendula are attached, see Fig. 7. On the other hand, *M*, *K*, 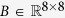 are the lumped mass, stiffness, and damping matrices, respectively. These matrices can easily be derived by following standard FE theory for beam elements, see e.g.^33^. The vector 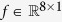 contains the external forces exerted by the pendula on the connecting nodes (node 2 and 3 in Fig. 7) and is given by


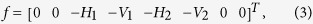


where


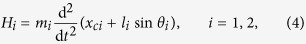



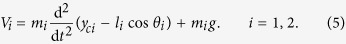


The remaining task is to model the escapement mechanism for the clocks. It is worth noting, however, that a proper model for the escapement is hard to obtain cf.^34^,^35^. This is the reason why some authors have modelled the escapement by using continuous^10^,^16^,^22^ and discontinuous^21^,^24^,^29^ nonlinear functions. These approximations seem to be sufficient to capture the essential behaviour of an escapement.

For the present case, the escapement mechanism of clock *i*, is modelled by the piece-wise linear function


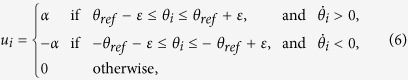


where 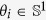, *i* = 1, 2, is the rotation angle of pendulum *i*, and *α*, *θ*_*ref*_, 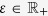 are design parameters.

The design of the scalar input *u*_*i*_, *i* = 1, 2, is very intuitive: each time the pendulum reaches a threshold angle, which is determined by *θ*_*ref*_ and *ε*, a step force of magnitude *α* is applied to the pendulum, either on the positive or negative direction, depending on the sign of the angular position and angular velocity. This behaviour coincides with the real operation of an anchor escapement in a clock: the anchor applies a small step force to the tooth of the escapement wheel when the angular displacement of the clock reaches a threshold angle, producing the characteristic ‘tic’ and ‘tac’ sounds. There are also instants at which the anchor escapement is not in contact with the escapement wheel. The interested reader is referred to^24^, where the operation of the proposed escapement (6) is illustrated.

By defining the state vector 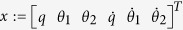, system (1)-(2) can be written in first order form


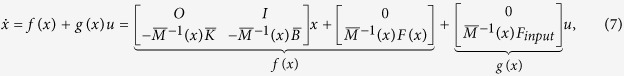


where *O* represents a matrix of zeros, 0 a vector of zeros and *I* the identity matrix, all of appropriate dimensions. 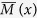, 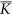, 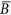 are the extended mass, stiffness, and damping matrices, respectively, *F* is a nonlinear vector, *F*_*input*_ is the input vector, and *u* = [*u*_1_ *u*_2_]^*T*^. These matrices and vectors are provided in Section Methods, see Eqs (25, 26, 27, 28, 29).

Note that the derived model contains the two essential components depicted on the right side of Fig. 1: a model for the coupling structure, which incorporates the flexibility properties of the structure, and the corresponding model for the clocks—including the escapement mechanism. Moreover, these two models are suitably coupled such that the influence of the clocks on the structure is contained in the term *f*, see Eq. (1), whereas the influence of the structure on the clocks is denoted by the first two terms on the right-hand side of Eq. (2).

Furthermore, at this point, the reader should be convinced that the resulting model (7) does not contain ‘artificial’ terms, since it has been derived following well established physical/mechanical laws. In other words, the model presented here seems to be the appropriate model for the experimental setup shown in Fig. 2.

A numerical analysis has been conducted with the final aim of reproducing the experimental results discussed before and, in this way, to validate our model. Consequently, system (7) with inputs (6) has been numerically integrated. The parameter values have been obtained from the experimental setup and are summarized in Table 1 and in Table 2, in Section Methods. Note that the pendula have been assumed to be identical. Additional details regarding the simulations are also provided in the aforementioned section.

**Figure 8:**
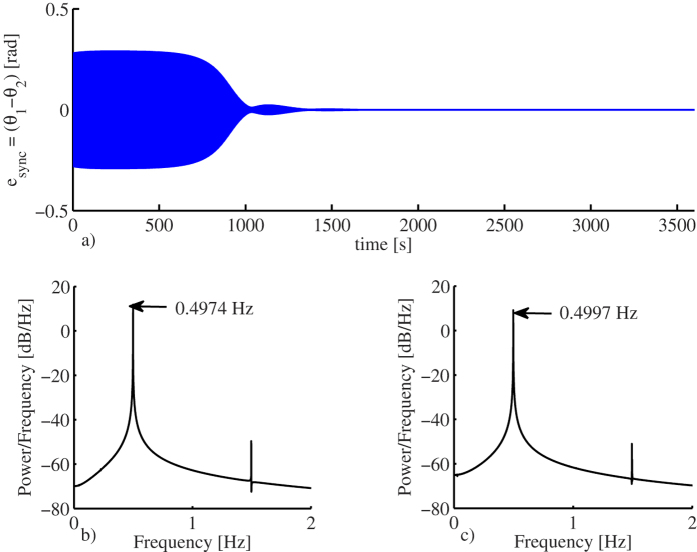
Simulation results for two identical pendulum clocks. (**a**) Synchronization error 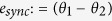. (**b**) Oscillation frequency corresponding to the coupled and synchronized pendula. (**c**) Oscillation frequency for an uncoupled pendulum.

Figure 8 shows the obtained simulation results. Similar to the experiments, the pendula are initially released from opposite directions. However, after approximately 25 minutes, the clocks converge to in-phase synchronized motion. This is clearly seen in Fig. 8(a), where the synchronization error 
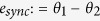
 is depicted. Note that the synchronization error vanishes completely, whereas in the experiment, see Fig. 4(d), the synchronization error remains within a region around zero, i.e. in the experiments the error remains small but never converges to zero. This result, however, is obvious because in the experiment, there are unavoidable mismatches between the clocks, whereas for the computer simulations the clocks have been assumed to be identical.

The simulation results also reveal that the oscillation frequency of the coupled and synchronized pendula is 0.4974 [Hz], whereas the oscillation frequency of an uncoupled pendula is 0.4997 [Hz], as depicted in Fig. 8(b,c). Hence, just like in the experiments, the pendula become slower.

In conclusion, by comparing the experimental results presented in Fig. 3 and the obtained numerical results depicted in Fig. 4, it is evident that the derived model (7) is able to capture the dynamical behaviour of the two coupled monumental clocks presented in Fig. 2.

### Analytic results

So far, the onset of in-phase synchronous motion in a pair of pendulum clocks has been demonstrated by means of experiments and computer simulations. The next step is to conduct an analytic study in order to determine when and under which conditions, the clocks will show *asymptotically stable* synchronized motion.

The analysis starts by assuming ‘small’ oscillations in the pendula, i.e. by considering that cos *θ*_*i*_ ≈ 1 and sin *θ*_*i*_ ≈ *θ*_*i*_. Note that this is indeed a mild assumption, since by a suitable adjustment/design of the escapement mechanism, it is possible to keep the amplitude of the oscillations in the pendulum of the clock within a small value.

If the assumption of small oscillations holds, then the dynamic behaviour of system (7) can be analyzed by linearizing the system around the origin. After linearization, system (7) with inputs (6) takes the form


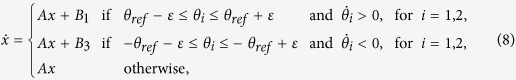


where 
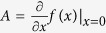
, *B*_1_ = *αg*(0) and *B*_3_ = −*αg*(0), with *f* and *g* as defined in (7) and *α* as given in (6).

System (8) is a piece-wise linear (PWL) system, with state dependent switching rule (remember that *θ*_*i*_ is indeed a state variable of the state vector *x* defined in (7)). Note that the PWL system (8) has 8 switching surfaces, i.e. hyperplanes at which the trajectory ‘jumps’ from one subsystem to another. However, when the pendula are synchronized, i.e. *θ*_1_ = *θ*_2_ and 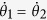, the number of switching surfaces reduces to 4. These surfaces are given by


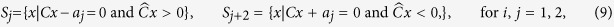


where *a*_1_ = *θ*_*ref*_ − *ε*, *a*_2_ = *θ*_*ref*_ + *ε*, and the row vectors *C* = [0_0_ 1 0 0_1_] and 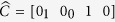, where 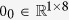 and 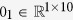 are row vectors of zeroes.

Under these considerations, it is possible to obtain sufficient and necessary conditions for the existence of an isolated synchronized periodic solution, i.e. a ‘synchronous limit cycle’ in the PWL system (8). The following proposition, which can be easily proved by following the results presented in^36^,^37^, provides such conditions.

**Proposition 1** *Consider the PWL system* (8). *Assume that there exists an isolated periodic solution*, *i.e. a limit cycle with* 4 *switches per cycle*, *as defined in* (9), *such that a trajectory starting from the switching surface S*_1_ *hits the switching surfaces S*_2_ *at time t*_1_. *Then*, *the trajectory moves forward from S*_2_ *to S*_3_ *in t*_2_ *seconds*, *from S*_3_ *to S*_4_ *in t*_3_ *seconds*, *and finally*, *from S*_4_ *back to S*_1_ *in t*_4_ *seconds*. *Hence*, *the period of the limit cycle solution is* 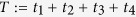. *Define*


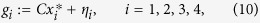



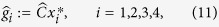


*where η*_1_ = −*a*_2_, *η*_2_ = *a*_1_, *η*_3_ = *a*_2_, *η*_4_ = −*a*_1_, *with a*_1_ *and a*_2_ *as given in* (9), *and* 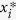 *are the values of the limit cycle solution at the switching surfaces* (9), *and are given by*


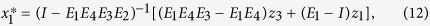



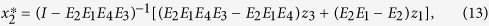



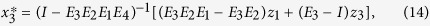



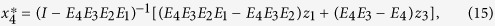


*with* 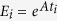 *for* i = 1, 2, 3, 4, *z*_*r*_ = *A*^−1^ *B*_*r*_, *for r* = 1, 3.

*Then the following conditions hold:*


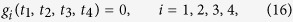



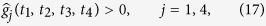



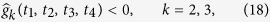


*and the periodic solution is governed by system* 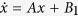 *in the interval* [0, *t*_1_), *by system* 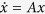 *in the interval* [*t*_1_, *t*_2_), *by system* 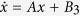 *in the interval* [*t*_2_, *t*_3_), *and again by system* 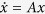 *in the interval* [*t*_3_, *t*_4_).

**Figure 9:**
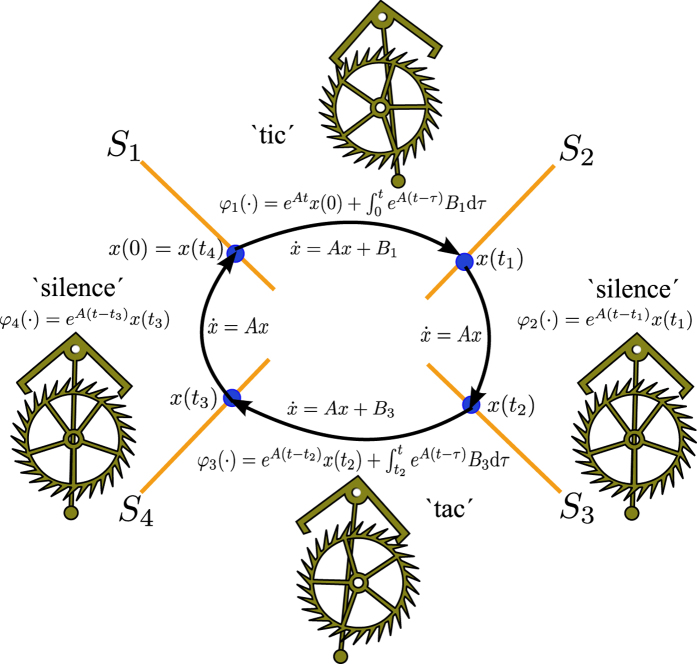
Limit cycle in the PWL system (8).

The limit cycle described in Proposition 1 is illustrated in Fig. 9. The trajectory starts from the switching surface *S*_1_ at *t* = 0, i.e. 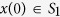. Then, system 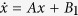 brings the trajectory to the switching surface *S*_2_ at time *t*_1_ by means of the mapping *φ*_1_(·). Thereafter, the trajectory is conducted by system 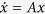 with initial condition 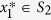 until the switching surface *S*_3_ is hit at *t*_2_. Next, the trajectory evolves according to 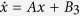, i.e. by means of the mapping *φ*_3_(·) the trajectory is driven to the switching surface *S*_4_ at time *t*_3_. Finally, the trajectory returns to the ‘departing’ switching surface *S*_1_ at time *t*_4_ via the mapping *φ*_4_, which is associated to system 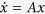. Since the operation of the escapement is symmetric, i.e. the ‘tic’ and ‘tac’ sounds are produced equidistantly in a clock, the times at which the switching surfaces are reached are also symmetric such that *t*_3_ = *t*_1_ and *t*_4_ = *t*_2_.

From a physical point of view, the limit cycle of Fig. 9 is explained as follows. Initially, the escapement wheel of the clock is in contact with the anchor escapement mechanism and remains in this situation in the interval (*t*_0_, *t*_1_). In this interval, the clock produces the characteristic ‘tic’ sound. Then, the escapement wheel is released from the anchor escapement and the pendulum of the clock oscillates freely, i.e. there is a ‘silence’ period in the interval (*t*_1_, *t*_2_) until the escapement wheel is again engaged at time *t*_2_. The anchor escapement remains in contact with the escapement wheel in the interval (*t*_2_, *t*_3_), producing the ‘tac’ sound. Once the escapement wheel is released at *t*_3_, the pendulum oscillates back to the starting point, i.e. there is again a silence period in the interval (*t*_3_, *t*_4_) and finally, the escapement wheel is engaged at *t*_4_, i.e. the cycle starts again.

Next, the asymptotic stability of the limit cycle described above is investigated by using the following proposition, which is based on a general result derived and proved in refs 36,37.

**Proposition 2** *Consider the PLS system* (8) *and assume that the system has a limit cycle as described in Proposition* 1. *Define*


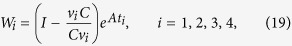


*where* 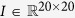 *is the identity matrix*, 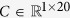 *is a row vector as defined in* (9), *matrix* 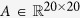 *as given in* (8), 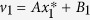, 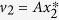, 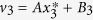 *and* 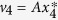, *with* 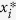, *i* = 1, 2, 3, 4, *as defined in* (12–15). *Moreover*, *assume that the limit cycle is transverse to the switching surfaces*, *i.e. Cv*_*i*_ ≠ 0. *Then*, *if all the eigenvalues of the matrix*


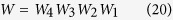


*are contained inside the unit circle*, *then the limit cycle described in Proposition* 1 *is locally stable and unstable otherwise*.

Based on the aforementioned propositions, the following result is true.

**Theorem 1** *Consider the PWL system* (8). *If it is possible to find the smallest times t*_1_, *t*_2_, *t*_3_ = *t*_1_ *and t*_4_ = *t*_2_, *satisfying conditions* (16–18) *in Proposition* 1 *and*, *simultaneously*, *satisfying*


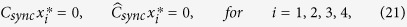


*where C*_*sync*_ = [0_0_ 1 −1 0_1_], 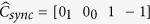 *and with* 0_0_ *and* 0_1_ *as defined in* (9), *then the following holds*


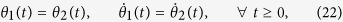


*i.e. the in-phase synchronous solution of the state variables θ*_*i*_, 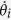, *i* = 1, 2, *which respectively describe the angular displacement and angular velocity of the coupled pendula*, *exists*. *Furthermore*, *the oscillation frequency of this synchronous solution is*


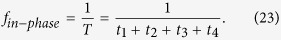


*Additionally*, *if for the obtained times t*_*i*_, *i* = 1, 2, 3, 4 *the eigenvalues of* (20) *are contained inside the unit circle*, *then the in-phase solution* (22) *is locally asymptotically stable*.

At this point, it should be noted that the values of *t*_1_, *t*_2_ and *t*_3_ = *t*_1_, *t*_4_ = *t*_2_ satisfying the conditions in Proposition (1) and the conditions in Theorem (1), cannot be obtained in closed form, in part because of the fact that the number of variables is smaller than the number of equations. The best we can do is to numerically solve (16) subject to conditions (17), (18), and (21).

**Remark 1** The analytic results have been derived for the PWL system (8). However, under the assumption of ‘small’ oscillations in the pendula, the obtained results hold for the original system (7). Hence, if the conditions of Theorem 1 are fulfilled, we may expect that the monumental clocks will synchronize in-phase with oscillation frequency approximately equal to (23). Moreover, such synchronized motion is expected to be locally asymptotically stable.

Finally, the obtained analytic results are used in order to investigate the onset of synchronization in the experimental setup of Fig. 2 with model (7). The smallest values for *t*_*i*_ satisfying Theorem 1 are obtained by solving (16) subjected to conditions (17), (18), and (21) and considering the parameter values given in Tables 1 and 2. This yields


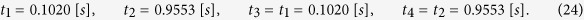


On the other hand, for the obtained times (24), the eigenvalues of (20) are all contained inside the unit circle. Hence, from Theorem 1, it follows that the in-phase synchronous motion in the coupled monumental clocks of Fig. 2 is expected to be locally asymptotically stable and the oscillation frequency of the synchronous solution is expected to be *f*_*in*–*phase*_ = 1/*T* = 1/(*t*_1_ + *t*_2_ + *t*_3_ + *t*_4_) = 1/2.1146 = 0.4729 [Hz]. These results are in good agreement with the experimental results, see Fig. 5 and with the numerical results, see Fig. 8. In fact, the errors between the obtained experimental and numerical results and the predicted analytic results remain lower than 5%.

## Discussion

The results presented in this manuscript provide new insight in understanding why two inert systems—monumental pendulum clocks— may feel sympathy for each other.

Specifically, we have demonstrated that two monumental pendulum clocks, placed on a wooden support, exhibit in-phase synchronized motion. Although the in-phase motion of pendulum-like oscillators and small pendulum clocks has already been reported, see e.g.^12^,^16^, this is the first time that the phenomenon is observed in monumental clocks. Additionally, we have discovered that the onset of synchronization has a cost namely, the oscillation frequency of the pendula decreases such that the clocks lose several seconds per hour. This result is different from Huygens’ experiment, where the clocks were oscillating in anti-phase. Moreover, in^22^ it has been demonstrated that in Huygens’ experiment, the oscillation frequency of the coupled and synchronized clocks is not affected.

**Figure 10:**
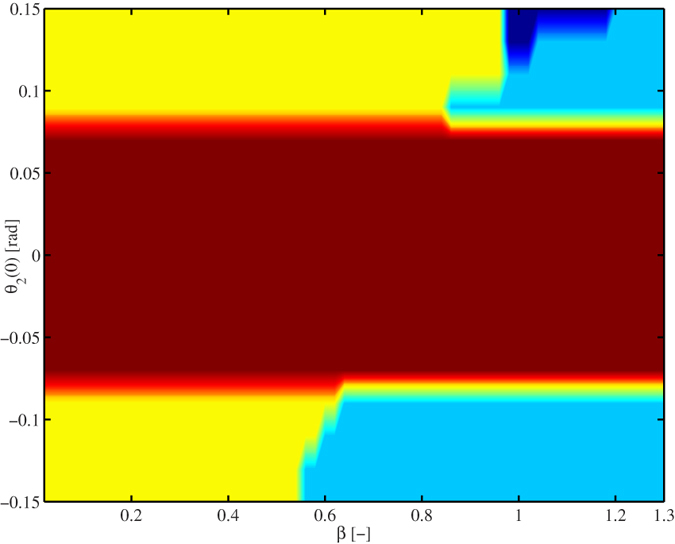
Limit behaviour of system (7) with input (6) as a function of the initial condition *θ*_2_(0) of pendulum two and the damping in the coupling structure, determined by *β*, see Eq. (30). The colors indicate the type of behaviour the system exhibits after 25000 [s]. Yellow: in-phase synchronization, cyan: anti-phase synchronization, brown: the initial condition of pendulum two is not enough to activate its escapement mechanism and consequently, pendulum two comes to stand still, blue: the pendula exhibit frequency synchronization, but the phase difference is neither 0 nor *π*. For all cases, the initial condition of pendulum one has been fixed to *θ*_1_(0) = 0.15 [rad].

At this point, the reader may be wondering why Huygens was observing anti-phase synchronization while in our experiment we have observed in-phase synchronization. In fact, there is an easy explanation: the mechanical properties of the structure used here are different than the mechanical properties of the setup used by Huygens. Moreover, the parameters of the pendulum clocks are also different from the ones used by Huygens. The reader should realize that there is not a unique key parameter but rather a set of key parameters, which determine the type of synchronization, e.g. damping and stiffness in the coupling structure, and mass, length, and damping in the pendula. The combination of all these parameters will eventually lead to a specific type of synchronous motion, to co-existence of synchronous solutions, or even to other kind of limit behaviour, see e.g.^24^. For the sake of illustration, we have included Fig. 10, which clearly shows that the damping in the coupling structure (related to parameter *β*, see Eq. (30) below, in Section ‘Methods’) plays a key role in the type of synchronization in the pendulum clocks. From this figure it is evident that for small damping (*β* < 0.55) only in-phase synchronization exists (yellow region). For large damping (*β* ≥ 1.195) anti-phase synchronization is the dominant behaviour (cyan region) and for ‘intermediate’ damping in the coupling structure (0.55 ≤ *β* ≤ 0.95) in-phase and anti-phase synchronization co-exist, depending on the initial conditions. Also note that for a small interval of the damping parameter (0.95 ≤ *β* ≤ 1.195) there exists a region where the pendula are synchronized in frequency but with a constant the phase difference, which is neither 0 nor *π* (blue region). Clearly, the results presented in Fig. 10 suggest that in the case of Huygens’ setup of coupled clocks the damping was ‘large’ since Huygens only observed anti-phase synchronization, whereas in the experimental platform presented here the damping is ‘small’ and consequently we only have observed in-phase synchronization cf.^9^,^12^.

Although to the best of our knowledge we have witnessed something that Huygens did not see—two pendulum clocks showing in-phase synchronized motion but becoming slow/inaccurate—it should be emphasized that this is not the end of the history. We believe that there are more limit behaviours ‘hidden’ in Huygens’ system of coupled clocks, see e.g.^24^. Then, the challenge—among others—is to find those limit behaviours not only at the level of computer simulations but also at the level of experiments and to rigorously prove their existence and stability. Moreover, although the mathematical model presented here is indeed an improved model regarding current models, the ‘true’ Huygens’ model is still ‘a mystery to unveil’. Consequently, we encourage Huygens’ followers to continue in these directions.

Finally, it is worth mentioning that the experimental setup depicted in Fig. 2 has been placed, for indefinite time, in the clocks museum “Alberto Olvera”. Everyday, dozens of visitors can admire the sympathetic motion of the clocks. It seems that for them, it is truly amazing—perhaps miraculous as some of them have commented—to observe a pair of inert systems oscillating in perfect consonance. A short text next to the sympathetic clocks reads ‘…*the pendulum clocks show sympathetic motion due to the imperceptible motion of the wooden support on which they are placed…this sympathy*, *however*, *prevents the clocks of being accurate…’*

## Methods

The dimensions and mechanical properties of the coupling structure, see Figs 2 and 7, are summarized in Table 1.

On the other hand, Table 2 summarizes the main characteristics of the pendulum clocks and the parameter values for the nonlinear function (6), which mimics the escapement mechanism of the clocks.

Note that Tables 1 and 2 summarize the parameter values for the ideal case, i.e. for the case of identical pendulum clocks and identical mechanical properties in the coupling structure. However, in the real experiment, the clocks are not identical although they have constructed as identical as possible. Here, we have assumed identical systems for the sake of easy analysis, see Subsection ‘Analytic results’. In fact, the parameter values presented in Table 2 correspond to pendulum one. The real parameter values of pendulum two are *m*_2_ = 5.12 [kg], *l*_2_ = 0.9940 [m] and *m*_*case*2_ = 30.3 [kg], just to mention a few. If the actual differences between the clocks are taken into account, then it is not trivial to perform an analytic study showing the robustness of the synchronized motion against mismatches between the pendulum clocks. Such analysis is still an open question, whose answer is beyond the scope of this manuscript. Nevertheless, we want to stress the fact that the ideal case considered here provide important insight in understanding the onset of in-phase synchronous solutions in the real system of coupled clocks depicted in Fig. 2.

In the experiments, the angular displacement of the pendula has been measured by means of magnetic sensors HCM1501, from Honeywell^38^. A magnet has been attached to the pendulum of each clock and the corresponding sensor has been placed in the case of the clock. Additionally, a data acquisition card from Texas Instruments, has been used for recording the data to a computer. For the simulation results, see Fig. 8, the derived model (7) with input (6) was implemented in Simulink and numerically integrated by using the variable time-step solver *ode*23*t*, available in MatLab©, with relative and absolute tolerances equal to 0.001 and maximum step size equal to 0.001. The initial conditions are *θ*_1_(0) = 0.1 [rad], *θ*_2_(0) = −0.096 [rad], and the remaining initial conditions are set to zero.

The matrices corresponding to model (7) are given by


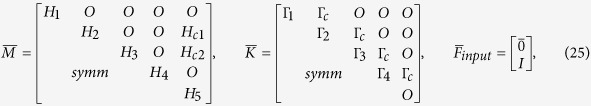


where 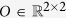, and 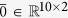 are zero matrices, 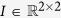 is the identity matrix, and the square matrices 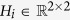, *i* = 1, …, 5, are given by


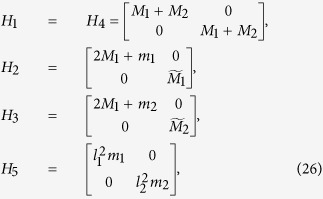


where *M*_1_ = *ρ*_*h*_*A*_*h*_*l*_*b*_/2, *M*_2_ = *ρ*_*v*_*A*_*v*_*l*_*S*_/2, 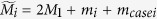, *i* = 1, 2, 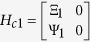, 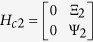 with 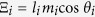, 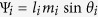, *i* = 1, 2, and


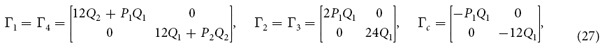


where 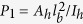, 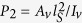, 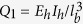, and 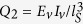.

On the other hand, the nonlinear vector 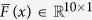 in (7) verifies


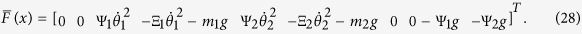


Finally, the damping matrix is given by^24^


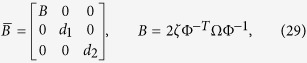


where *ζ* is the dimensionless damping coefficient, 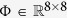 contains the eigenmodes *φ*_*i*_, which are obtained by solving the eigenvalue problem 
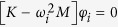
, for *i* = 1, …, 8, with *M* and *K* as given in (1). Moreover, the eigenmodes *φ*_*i*_ have been normalized such that Φ^*T*^*M*Φ = *I*, where 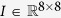 is the identity matrix. Likewise the eigenmodes satisfy Φ^*T*^*K*Φ = Ω^2^, where 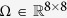 is a diagonal matrix containing the eigenfrequencies *ω*_*i*_ of the coupling structure.

In order to generate Fig. 10, we have assumed that the damping matrix *B* associated to the structure is proportional to the stiffness matrix, i.e. we have considered that


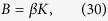


where 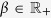 and *K* as defined in (1). Again, the derived model (7) with input (6) and parameter values as summarized in Tables 2 and 1 was implemented in Simulink and numerically integrated by using the variable time-step solver *ode*23*t*, available in MatLab©, with relative and absolute tolerances equal to 1*e* − 5 and automatic maximum and minimum step size. The initial conditions are *θ*_1_(0) = 0.15 [rad], *θ*_2_(0) is varied in the interval [−0.15, 0.15] [rad], in steps of 0.25 [rad] and the remaining initial conditions are set to zero. The proportional damping parameter *β* is varied in the interval [0.02, 1.3] in steps of 0.02.

## Epilogue

Nowadays, there exists a large number of studies addressing the phenomenon of Huygen’s synchronization. This may be motivated by the fact that Huygens’ system of coupled clocks has a certain degree of similarity with other systems.

Consider for instance the case of two driven unbalanced rotors (a familiar example of this kind of devices is a washing machine) mounted on an elastic support, i.e. interacting via Huygens’ coupling. It has been found that, under certain conditions, the rotors may synchronize either in-phase or in anti-phase^16^. Note that the onset of anti-phase synchronization in this example is highly desirable, since this will reduce or even eliminate the vibrations of the common support during the operation of the rotors. However, the onset of in-phase synchronization is not desired at all, since this will induce resonance and high amplitude vibrations of the support ultimately resulting in harmful and undesirable effects.

Something similar happens in a living organism. For instance, inside the human body, there are several biological rhythms: respiration, heartbeat, and blood perfusion just to mention a few of them. It has been found that when some of these rhythms synchronize with each other the energy consumption is minimal^39^,^40^. Hence, in this case the onset of synchronization is beneficial. On the other hand, synchronization can also be dangerous or detrimental. It is widely accepted, that the process of seizure generation is closely associated with abnormal synchronization of neurons, see e.g.^41^.

Note that in both cases, either unbalanced rotors or biological rhythms in the human body, the synchronization phenomenon occurs naturally. Therefore, it is necessary to determine under which conditions (maybe related to the coupling structure) the synchronization phenomenon (in particular, in-phase or anti-phase) may occur. This suggests that perhaps the (theoretical and/or experimental) achievements in one area, say mechanics, can help to better understand the natural synchronization phenomena occurring in for instance biological rhythms, where a rigorous theoretical study is most of the time unfeasible because of the obvious lack of good mathematical models.

Moreover, Huygens’ synchronization also finds interesting applications. For example, in cancellation of vibrations^42^ and in determining the behaviour of coupled transmission lines, cf.^43^. Additionally, it should be noted that there is a connection between the synchronization phenomena like the one observed by Huygens and a phenomenon which is referred to as indirect synchronization^44^,^45^,^46^,^47^,^48^.

Hence, it should be clear that Huygens’ setup of coupled pendulum clocks is an exciting and challenging nonlinear dynamical system, whose dynamical behaviour is far from being completely understood. Further studies of this system will lead to unveiling more details about the complex yet intriguing synchronization phenomenon.

Last but not least, we want to comment the following. Recently, the sympathetic motion of pendulum clocks has been the topic of several newspapers and magazines. The reason: a research paper, in which the authors claim that the synchronization between clocks (Huygens’ synchronization) takes place due to sound pulses (sound solitons)^30^. It is our strong believe that when reading our paper, and the results presented by other ‘Huygens’ followers’, see e.g.^9^,^12^,^20^,^21^,^23^,^29^, the reader will be convinced that the ‘secret’ behind Huygens’ synchronization is enclosed into the dynamics of the coupling structure on which the clocks are hanging. In other words, the reader should realize that if the clocks are suitably coupled, they will synchronize even if the sound pulses are absent.

## Additional Information

**How to cite this article**: Peña Ramirez, J. *et al.* The sympathy of two pendulum clocks: beyond Huygens’ observations. *Sci. Rep.* **6**, 23580; doi: 10.1038/srep23580 (2016).

## Figures and Tables

**Table 1:** Geometrical and material (wood) properties for the coupling structure.

Property	Horizontal beam	Each vertical beam
Length	*l*_*B*_ = 0.84 [m]	*l*_*S*_ = 1 [m]
Width	*b*_*h*_ = 0.40 [m]	*b*_*v*_ = 0.04 [m]
Thickness	*h*_*h*_ = 0.16 [m]	*h*_*v*_ = 0.04 [m]
Cross-section area	*A*_*h*_ = *b*_*h*_*h*_*h*_ = 0.064 [m^2^]	*A*_*v*_ = *b*_*v*_*h*_*v*_ = 0.0016 [m^2^]
Second moment of area	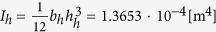	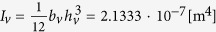
Density	*ρ*_*h*_ = 770 [kg/m^3^]	*ρ*_*v*_ = 770 [kg/m^3^]
Young’s modulus	*E*_*h*_ = 8.963 · 10^9^ [N/m^2^]	*E*_*v*_ = 8.963 · 10^9^ [N/m^2^]
Other dimensions and parameters, see Fig. 7.		
Length for a single horizontal beam after discretization	*l*_*b*_ = 0.28 [m]	
Damping coefficient in the structure, see (29)	*ζ* = 1[−]	
Gravitational acceleration	*g* = 9.81 [m/s^2^]	

**Table 2:** Parameter values for the ideal pendulum clocks used in the numerical analysis.

Property	Pendulum one	Pendulum two
Mass of the pendulum bob	*m*_1_ = 5 [kg]	*m*_2_ = 5 [kg]
Mass of the metallic clock case	*m*_*case*1_ = 30 [kg]	*m*_*case*2_ = 30 [kg]
Length of pendulum	*l*_1_ = 0.99 [m]	*l*_2_ = 0.99 [m]
Damping at the revolute joint	*d*_1_ = 0.065 [Nms/rad]	*d*_2_ = 0.065 [Nms/rad]
Parameter values for the escapement mechanism, see (6)		
Amplitude of the applied input	*α* = 0.1660 [Nm]	
Threshold angle	*θ*_*ref*_ = 0.0750 [rad]	
‘Deviation’ angle	*ε* = 0.010 [rad]	
